
JOURNAL INFORMATION
==============================
NLM Title Abbreviation: ScientificWorldJournal
Journal ID: ScientificWorldJournal
NLM Journal ID: 101131163
Journal ID: TSWJ

The Scientific World Journal

ISSN: 2356-6140
EISSN: 1537-744X
Publisher: Wiley

ARTICLE INFORMATION
==============================
PMCID: PMC6084714
PMID: 30147600
DOI: 10.1100/tsw.2001.220
Article ID: 985924
Article version: 1
Subjects: Short Report

Tissue Inhibitor of Metalloproteinase-1 Inhibits Apoptosis of Rat and Human Hepatic Stellate Cells In Vitro

Murphy Frank R. *
Issa Razao
Benyon Christopher
Iredale John P.
Liver Group, Cell & Molecular Medicine, Southampton University, Southampton, Hampshire SO16 6YD, UK
*Frank R. Murphy: frm105@hotmail.com
Publication date: 2001
Electronic publication date: 2001 Oct 18
Volume: 1
First page: 119
Last page: 119
Copyright: Copyright © 2001 Frank R. Murphy et al.
Copyright year: 2001
License: This is an open access article distributed under the Creative Commons Attribution License, which permits unrestricted use, distribution, and reproduction in any medium, provided the original work is properly cited.
License URL: https://creativecommons.org/licenses/by/3.0/

==============================
Miami *Nature Biotechnology* Short Reports
*TheScientificWorld* (2001) 1 (S3), 119SR
ISSN 1532-2246; DOI 10.1100/tsw.2001.220

### TISSUE INHIBITOR OF METALLOPROTEINASE-1 INHIBITS APOPTOSIS OF RAT AND HUMAN HEPATIC STELLATE CELLS IN VITRO

*Frank R Murphy, Razao Issa, Christopher Benyon, John P. Iredale

Liver Group, Cell & Molecular Medicine, Southampton University, Southampton, Hampshire SO16 6YD, U K
*frm105@hotmail.com

**INTRODUCTION.** Liver fibrosis represents the final common pathway for the majority of chronic liver insults (alcohol, autoimmune or viral injury). Current evidence indicates that the central mediator of fibrosis is the hepatic stellate cell (HSC). During fibrotic injury, these retinoid rich cells proliferate and undergo a phenotypic transformation to myofibroblast-like cells, a process termed activation. Previous work by the our group has demonstrated that activated HSC also express the powerful tissue inhibitors of metalloproteinases (TIMPs) 1 and 2, suggesting that matrix degradation is inhibited during fibrogenesis. This hypothesis is supported by findings that spontaneous recovery from liver fibrosis is associated with a diminution of TIMP expression and an increase in collagenase activity with consequent matrix degradation (1). A further finding in this study is that sustained apoptosis of HSC accompanies recovery from fibrosis. This has highlighted the role of TIMP-1 and -2 in HSC proliferation, survival and apoptosis.

**AIMS.** To determine the effect of TIMP-1 on human and rat hepatic stellate cell proliferation and apoptosis.

**METHODS.** Human HSC were extracted from the margins of normal human liver resected for colonic metastatic disease. Rat HSC were extracted from normal rat liver by pronase and collagenase digestion and purified by centrifugal elutriation as described (2). Extracted HSC were cultured in 15% fetal calf serum on plastic until they were activated to a myofibroblastic phenotype. The response of HSC in terms of proliferation and apoptosis was measured by tritiated thymidine incorporation, acridine orange staining, Caspase-3 activity and TUNEL staining. To control for the effect of matrix metalloproteinase inhibition, a synthetic matrix metalloproteinase inhibitor (BB-94) was also used.

**RESULTS.** TIMP-1 at concentrations of 1-200ng/ml had no effect on proliferation of rat HSC (n=4) over a 24 hr incubation period. However, assessment of nuclear morphology following acridine orange staining showed that a 4 hr incubation with TIMP-1 significantly reduced apoptosis of human and rat HSC induced by cycloheximide in a dose dependent manner (n=5, $p<0.0001$) at a dose range of 1-200ng/ml. A similar effect was observed after a 24-hour incubation in serum free conditions. Bovine serum albumin, used as a carrier for the TIMP-1, had no anti-apoptotic effect at the same concentration. In parallel studies, the synthetic matrix metalloproteinase inhibitor BB-94 (5-10uM) had no protective effect, suggesting that the anti-apoptotic effect of TIMP-1 was independent of matrix metalloproteinase inhibition. This anti-apoptotic effect of TIMP-1 was also supported by studies using a colorimetric assay for Caspase-3 activity and TUNEL staining.

**DISCUSSION.** During recovery from liver fibrosis in the rat carbon tetrachloride model of injury there is a diminution of HSC number mediated by apoptosis. At the same time there is a reduced expression of TIMP-1. Our results indicate that there may be a mechanistic association between changes in TIMP-1 and HSC numbers in recovery as we show that TIMP-1 has a significant and dose dependent anti-apoptotic effect on both human and rat HSC in vitro. This may prove to be an important therapeutic target in the treatment of liver cirrhosis in the future.

**CONCLUSION.** TIMP-1 has an anti-apoptotic effect on human and rat hepatic stellate cells that is dose dependent and is independent of matrix metalloproteinase inhibition.

**ACKNOWLEDGEMENTS.** The authors gratefully acknowledge the support of the MRC UK. JPI is an MRC Senior Clinical Fellow and a member of an MRC COGG. RI is a Hope Fellow sponsored by the Wessex Medical Trust. FRM is an MRC Clinical training fellow.

**REFERENCES.**

1. Iredale, J.P. et al. (1998) J. Clin. Invest. 102(3), 538-549
2. Knook, D.L. and DeLeeuw, A.M. (1982) Sinusoidal Liver Cells. Elsevier Biomedical Press, pp 45-52
